# Spiral Humeral Fracture Sustained During an Arm-Wrestling Match: A Case Report

**DOI:** 10.7759/cureus.105857

**Published:** 2026-03-25

**Authors:** Alec Mirchandani, James Espinosa, Alan Lucerna

**Affiliations:** 1 Emergency Medicine, Jefferson Health, Stratford, USA

**Keywords:** arm wrestling injury, spiral fracture of humerus from arm wrestling, spiral humeral fracture, torsional injury to humerus, upper extremity trauma

## Abstract

Humeral fractures are relatively uncommon, with spiral patterns typically associated with torsional forces. Arm wrestling is an activity that can lead to humeral shaft fractures. This report presents the case of a 25-year-old male who sustained a spiral fracture of the right humerus during an arm-wrestling match. Radiographic evaluation confirmed the diagnosis, and the injury was successfully managed with closed reduction followed by immobilization using a posterior long arm splint. This case underscores the need to consider atypical mechanisms of injury, such as arm wrestling, when evaluating humeral fractures. Additionally, it supports the efficacy of conservative, non-operative management in young, otherwise healthy patients with isolated humeral fractures.

## Introduction

Humerus fractures, particularly spiral fractures, are typically caused by rotational or twisting forces. Arm wrestling is a worldwide sport practiced by both men and women [1]. Arm wrestling has been traced as far back in history as ancient Egypt, and formal competition began in the 1950s in the United States [2].

In this case, the mechanism of injury during arm wrestling is consistent with this type of fracture. Arm wrestling involves intense flexion and torque, often causing a rotational force on the upper arm that can lead to a spiral fracture of the humerus [3,4]. The most common fracture seen with arm wrestling is a humeral spiral fracture. However, other injuries have been reported, including scapular neck fractures, anterior dislocation of the elbow, and other upper extremity injuries [1-3].

This case report was presented in poster form at the Rowan-Virtua Research Day, May 8, 2025, Stratford, NJ, USA.

## Case presentation

A 25-year-old male presented to the emergency department (ED) with acute right upper arm pain sustained during an arm-wrestling match. The patient reported a sudden onset of severe pain following forceful exertion against his opponent.

On examination, the patient was alert and oriented but in moderate distress due to pain. Vital signs were within normal limits: temperature 98.8°F (37.1°C), heart rate 90 beats per minute, respiratory rate 16 breaths per minute, and blood pressure 128/72 mmHg. Inspection of the right upper extremity revealed visible deformity localized to the proximal region. Palpation elicited marked tenderness over the lateral aspect of the distal humerus. The patient was unable to actively mobilize the limb due to pain. There were no open wounds or signs of skin tenting. Distal neurovascular status was intact, with preserved sensation and 2+ palpable radial pulses. The remainder of the physical examination was unremarkable.

**Figure 1:**
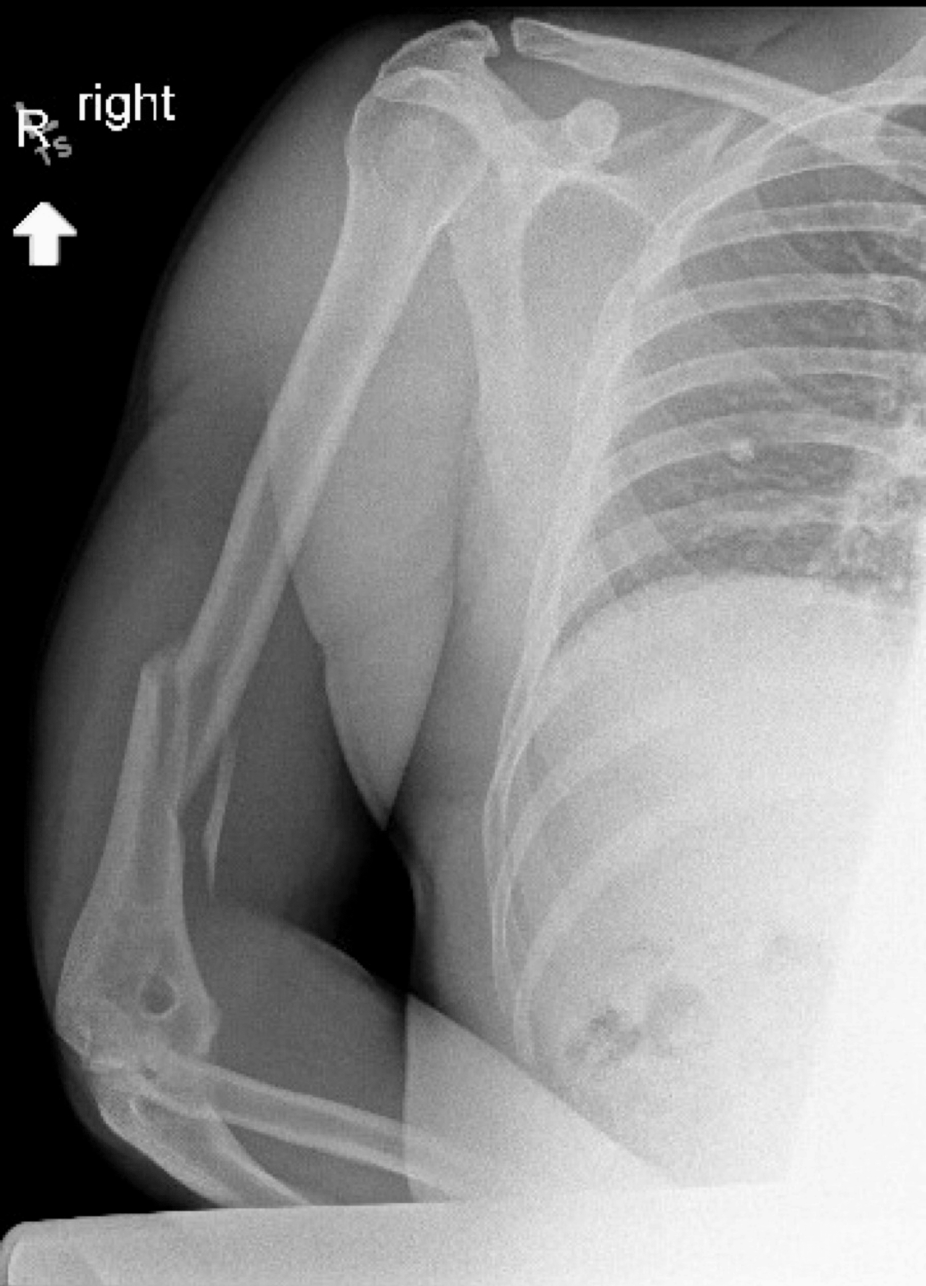
Comminuted, displaced, angulated distal humeral diaphyseal fracture

The patient had no significant past medical history, including no previous fractures or known musculoskeletal disorders. Plain radiographs of the right upper extremity demonstrated a spiral fracture of the distal third of the humeral shaft with mild displacement (Figure 1).

**Figure 2:**
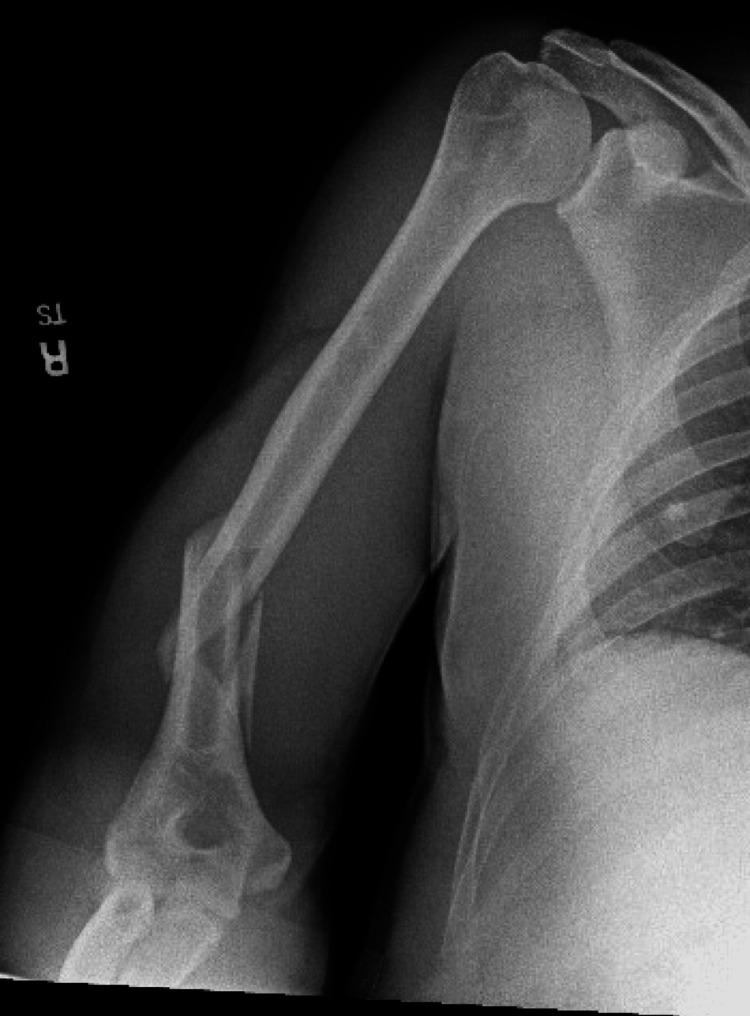
Improved alignment of the distal humeral diaphyseal fracture with a posterior long arm splint in place

In the absence of neurovascular compromise and given the patient’s age and the relatively stable nature of the fracture, conservative management was deemed appropriate. The orthopedic service was consulted, and closed reduction by the ED team was recommended. Intravenous analgesia was provided with 1 mg of hydromorphone. The patient underwent closed reduction in the supine position, with gentle manipulation yielding improved alignment. Post-reduction radiographs confirmed acceptable positioning with minimal residual angulation. The orthopedic service also felt that the post-reduction position was acceptable (Figure 2).

A posterior long arm splint was applied to immobilize the extremity. The patient was discharged with instructions to maintain non-weight-bearing status on the affected limb and to follow up with orthopedic services for continued evaluation and definitive outpatient management.

## Discussion

In a systematic review by Ogawa et al. of 153 humeral shaft fractures sustained during arm wrestling, all of the patients in which match details were recorded sustained the injury in association with a sudden exertion of intense flexion and torque in an attempt to change the match status [1]. This was the case in the patient presented. The fracture site is the lower third of the humerus, as was seen in this case, in 90% of cases [1,3]. Ogawa et al. found that almost 20% of cases had evidence of initial radial nerve palsy, with spontaneous recovery in 100% of cases [1]. Kim et al. found a similar rate of nerve palsy with noticeable recovery within five to seven months after the injury [5]. The high rate of recovery is felt to be associated with the lower energy of trauma in arm wrestling in comparison to blunt trauma from falls or motor vehicular accidents [6]. Shen et al. found no cases of radial nerve palsy in a series of 27 such injuries [7]. No radial nerve palsy was seen in this patient. Use of alcohol was noted to have been used in approximately 50% of the cases in the systematic review, suggesting that alcohol inebriation is not a major factor [1]. There was no history of alcohol use in the patient presented in this case. The relation of the development of such a fracture to the duration of the match has not yet been elucidated [3]. Amateurs involved in such matches are more prone to such injuries, perhaps due to stabilization of the arm at the level of the shoulder rather than by the placement of the elbow on the table [8]. The reason for the predominant distal humerus location may be due to a relatively thinner cortex in the distal humerus compared to the upper two-thirds of the humerus [4]. Men are more likely to sustain such fractures [9].

While the mechanism of injury is less common in everyday practice, it is important for clinicians to consider arm wrestling as a potential cause of humeral fractures, especially in young, active individuals. Spiral fractures are characterized by a twisting motion that creates a helical fracture pattern. These fractures are less likely to be displaced than transverse or oblique fractures, but they still pose a risk for complications such as nonunion or malunion [8].

In this case, the young age of the patient, the lack of significant displacement, and the absence of neurovascular injury contributed to the decision to proceed with closed reduction and non-operative management [4]. The management of humeral fractures in young individuals generally favors conservative treatment, especially in non-displaced or minimally displaced fractures. Closed reduction and casting have been shown to yield excellent outcomes, with high rates of healing and functional recovery [3,7].

Surgical intervention is typically reserved for fractures with significant displacement, open fractures, or those associated with neurovascular injury [10]. This case also emphasizes the importance of early mobilization and physical therapy following fracture healing. The goal of rehabilitation is to restore function and prevent stiffness or weakness, particularly in athletes such as this patient, who relies on upper limb strength.

## Conclusions

Fractures of the humerus sustained during arm wrestling are most commonly found in the distal third of the humerus and are spiral in nature. The nature of the forces operating on the humerus during arm wrestling, in combination with the relatively thinner nature of the bone of the distal third of the humerus, accounts for the location of the injury. The management of humeral fractures in young individuals generally favors conservative treatment, especially in non-displaced or minimally displaced fractures. Closed reduction and casting have been shown to yield excellent outcomes in such cases, with high rates of healing and functional recovery. This case highlights the need to maintain a high index of suspicion for humeral shaft fractures in patients presenting with arm pain following arm wrestling.
